# Construction of a Tetracycline Degrading Bacterial Consortium and Its Application Evaluation in Laboratory-Scale Soil Remediation

**DOI:** 10.3390/microorganisms8020292

**Published:** 2020-02-20

**Authors:** Xueling Wu, Yichao Gu, Xiaoyan Wu, Xiangyu Zhou, Han Zhou, Charles Amanze, Li Shen, Weimin Zeng

**Affiliations:** 1 School of Minerals Processing and Bioengineering, Central South University, Changsha 410083, China; wxlcsu@csu.edu.cn (X.W.); gyc951122@csu.edu.cn (Y.G.); wuxiaoyan1005@126.com (X.W.); zxy591868@163.com (X.Z.); zhhlzl@csu.edu.cn (H.Z.); charles.amanze@csu.edu.cn (C.A.); lishen@csu.edu.cn (L.S.); 2 Key Laboratory of Biometallurgy of Ministry of Education, Central South University, Changsha 410083, China

**Keywords:** Biodegradation of tetracycline, construction of bacterial consortium, lab-scale soil remediation, high-throughput sequencing, tetracycline resistance genes, mobile gene elements

## Abstract

As an environmental pollutant, tetracycline (TC) can persist in the soil for years and damage the ecosystem. So far, many methods have been developed to handle the TC contamination. Microbial remediation, which involves the use of microbes to biodegrade the pollutant, is considered cost-efficient and more suitable for practical application in soil. This study isolated several strains from TC-contaminated soil and constructed a TC-degrading bacterial consortium containing *Raoultella* sp. XY-1 and *Pandoraea* sp. XY-2, which exhibited better growth and improved TC degradation efficiency compared with single strain (81.72% TC was biodegraded within 12 days in Lysogeny broth (LB) medium). Subsequently, lab-scale soil remediation was conducted to evaluate its effectiveness in different soils and the environmental effects it brought. Results indicated that the most efficient TC degradation was recorded at 30 °C and in soil sample Y which had relatively low initial TC concentration (around 35 mg/kg): TC concentration decreased by 43.72% within 65 days. Soil properties were affected, for instance, at 30 °C, the pH value of soil sample Y increased to near neutral, and soil moisture content (SMC) of both soils declined. Analysis of bacterial communities at the phylum level showed that *Proteobacteria*, *Bacteroidetes*, *Acidobacteria*, and *Chloroflexi* were the four dominant phyla, and the relative abundance of *Proteobacteria* significantly increased in both soils after bioremediation. Further analysis of bacterial communities at the genus level revealed that *Raoultella* sp. XY-1 successfully proliferated in soil, while *Pandoraea* sp. XY-2 was undetectable. Moreover, bacteria associated with nitrogen cycling, biodegradation of organic pollutants, soil biochemical reactions, and plant growth were affected, causing the decline in soil bacterial diversity. Variations in the relative abundance of tetracycline resistance genes (TRGs) and mobile gene elements (MGEs) were investigated, the results obtained indicated that *tetD*, *tetG*, *tetX,* *intI1*, *tnpA-04*, and *tnpA-05* had higher relative abundance in original soils, and the relative abundance of most TRGs and MGEs declined after the microbial remediation. Network analysis indicated that *tnpA* may dominate the transfer of TRGs, and *Massilia*, *Alkanibacter*, *Rhizomicrobium*, *Xanthomonadales*, *Acidobacteriaceae*, and *Xanthomonadaceae* were possible hosts of TRGs or MGEs. This study comprehensively evaluated the effectiveness and the ecological effects of the TC-degrading bacterial consortium in soil environment.

## 1. Introduction

Tetracycline (TC) is massively used in livestock husbandry, serving as a feed additive or medication to treat diseases and infections caused by pathogenic microorganisms. Though the widespread application of TC benefits the related industries, a great risk is posed to the environment. TC cannot be fully metabolized by livestock, approximately 65% of the drug will be released into the environment in the form of parent compound or derivatives, causing contamination to edatope and water bodies. Several surveys have already been conducted to investigate TC contamination by former research. Qiao et al. detected TC pollutants of up to 15 mg/kg in soil collected from Putian, China [1], and Zhu et al. also detected TC pollutants with an average concentration of 15 mg/kg in soil contaminated by pig manure [2]. Moreover, TC pollutant is likely to be washed into the water environment by rains and overland runoff, causing extensive environmental issues. Investigations of TC contamination on catchment-scale were reported as well [3].

TC can linger in edatope for over 1 year, which is relatively longer than other antibiotics. The long persistence of the TC pollutant could cause various negative environmental effects, like reducing the diversity of indigenous microbial communities [4,5], enriching TC-resistant microbes, and damaging functional microbiota [6]. Additionally, the long presence of antibiotics will cause dissemination of the corresponding antibiotic resistant genes (ARGs), and human pathogenic microbes may acquire antibiotic resistance via mobile gene elements (MGEs)-mediated horizontal gene transfer, subsequently threatening human health. According to previous studies, tetracycline resistance genes (TRGs) were found to be the most prevalent ARGs in agricultural soils in China [7]. For instance, Zhu et al. detected 22 TRGs in soils near pig farms, and *tetG*, *tetL*, *tetA*, and *tetW* exhibited high abundance [2]. Moreover, it was believed that the abundance of TRGs is positively correlated with the concentration of TC in the environment, implying that heavily contaminated areas poses high ecological risks [8,9]. Hence, it is imperative to handle the issue of TC contamination.

So far, many methods have been developed to treat TC pollutants, including oxidative processes, such as ozonation (O_3_/H_2_O_2_), photocatalysis (UV/TiO_2_), ultraviolet (UV/H_2_O_2_), Fenton (Fe^2+^/H_2_O_2_), and adsorption treatment using different materials. Though some of these approaches have been successfully applied in TC treatment, the application scenarios are limited to the water environment. Very few technologies were invented to treat TC pollutant in edatope. Hitherto, some researchers found that TC degradation in soil could be accelerated by enhancing microbial degradation. For instance, the addition of biochar could change soil properties and increase the bioavailability of TC, thus improving the TC-degrading efficiency by microbes [10]. Additionally, composting of TC-contaminated sludge was investigated [11], and results indicated that increased bioactivity of TC-degrading microbiota promoted the TC degradation in soil. Thus, it can be seen that microbial methods are more applicable in the soil environment. From our perspective, it would be better to directly introduce high-efficient TC-degrading microbes to soil than to adopt other methods to enhance microbial degradation. As reported by previous researchers, certain bacterial isolates from contaminated environment could exhibit the potential to biodegrade antibiotic pollutants. Shao et al. found that *Ochrobactrum* sp. strain KSS10 had an outstanding ability to biodegrade oxytetracycline [12], and Leng et al. obtained a novel strain *Stenotrophomonas maltophilia* DT1, which was recorded to biodegrade 89% of 50 mg/L TC in LB medium [13]. Though the microbial degradation of antibiotics was proved to be effective, most applications were carried out in a liquid medium, and few studies investigated the TC-degrading effectiveness of microbes in the soil environment. In addition, not only should the effectiveness be considered, but attention also needs to be attached to the consequent environmental impacts as the introduction of foreign microbes is likely to affect the indigenous microbiota, and the application of TC-degrading bacteria may cause the dissemination of TRGs.

This study aimed to construct a TC-degrading bacterial consortium using bacteria isolated from contaminated soil, and its effectiveness on TC degradation would be evaluated via HPLC under the lab-scale soil system comprised of real TC-contaminated samples. Besides, influences generated by the introduction of the bacterial consortium on edatope would be comprehensively investigated, including the evolution of soil bacterial communities, changes of soil properties, and variations in TRGs and MGEs.

## 2. Materials and Methods

### 2.1. Chemicals and Medium

Lysogeny broth (LB) medium used for microbial cultivation consists of 10 g/L tryptone, 5 g/L yeast extract, and 5 g/L NaCl, the pH of which was adjusted to 7.0–7.2. The LB medium was autoclaved at 121 °C for 20 min. Mineral medium includes 15 mg/L EDTA solution, 4.5 mg/L ZnSO_4_∙7H_2_O, 4.5 mg/L CaCl_2_∙2H_2_O, 3 mg/L FeSO_4_∙7H_2_O, 1 mg/L MnCl_2_∙4H_2_O, 0.4 mg/L Na_2_MoO_4_∙2H_2_O, 0.3 mg/L CuSO_4_∙5H_2_O, 0.1 mg/L KI, 5 g/L (NH_4_)2SO_4_, 3 g/L KH_2_P0_4_, 0.5 g/L MgS0_4_∙7H_2_O (pH = 7.0–7.2), to which 18 g/L agar was added. After the same sterilization process, 50 mg/L TC was added before the medium solidified to prepare a single carbon source medium for strain isolation.

All chemicals used in this research were purchased from Sinopharm Chemical Reagent Co. Ltd. In addition to those mentioned above, the remaining are as follows: TC standard, chromatography grade methanol and acetonitrile, analytical grade sodium dihydrogen phosphate, disodium ethylenediaminetetraacetate, and citric acid.

### 2.2. Sampling and Isolation of Tetracycline (TC)-Degrading Bacteria

Two soil samples were collected from a hoggery in Changsha, where TC was used as a feed additive. Sample B was obtained from the site close to the sewage outlet, and sample Y was dug from the topsoil covered by pig feed. Both samples were stored at −20 °C for further studies. Isolation of TC-degrading bacteria was conducted as follows: soil suspension was prepared by dissolving 0.1 g soil in 10 mL sterilized water, then 1 mL supernatant was added into the LB medium containing 20 mg/L TC. The samples were then incubated at 30 °C in a dark shaker operated at 150 rpm for 3 days. Afterwards, 1 mL bacterial solution was inoculated into a new LB medium with 40 mg/L TC, and the inoculation continued for 3 more days with unchanged experimental conditions. The procedures above were repeated until the final TC concentration reached 180 mg/L. The diluted bacterial solution was daubed on Petri dishes made of a single carbon source medium. Several colonies with distinct morphology were isolated after a continuous purification process. Then, the growth curve assay was performed on the obtained bacteria in the mineral medium with 50 mg/L TC, and several biochemical analyses of bacteria were conducted as well. Lastly, the strains were cultivated separately in LB medium with 50 mg/L TC for further studies.

### 2.3. Construction of TC-Degrading Bacterial Consortium

Two strains (Accession numbers provided by the NCBI database: MN428656, MN428657) capable of biodegrading TC were isolated and they were named *A. faecalis* S-1 and *A. faecalis* S-2, respectively. In order to construct a TC-degrading bacterial consortium, another two strains were collected from the culture collection of the laboratory, both of which were isolated from different TC-contaminated soil. One is *Raoultella* sp. XY-1 (Accession number provided by the NCBI database: MN428658), and the other is *Pandoraea* sp. XY-2 (CP030849) [14]. For convenience, the four strains were abbreviated as follows: S1 stands for *A. faecalis* S-1, S2 stands for *A. faecalis* S-2, R stands for *Raoultella* sp. XY-1, and P stands for *Pandoraea* sp. XY-2. Several bacterial consortia were constructed using different combinations of the four strains, including S1&S2, S1&P, S1&R, S2&P, S2&R, P&R, S1&S2&P, S1&S2&R, S1&P&R, S2&P&R, and S1&S2&P&R. The combination ratio of each strain is 1:1. A further experiment was carried out in 150 mL LB medium with 50 mg/L TC, pH = 7.0, and the shake flasks were set on a dark shaker set at 30 °C, and 150 rpm. The inoculation volume of bacterial suspensions was 3 mL. All experiments were triplicated, including control groups without adding TC-degrading bacteria. The concentration of TC was successively quantified via HPLC at different cultivation timing (i.e., 1st, 2nd, 3rd, 4th, 5th, 6th, 7th, 8th, 10th, 12th, 14th day), and optical density (OD) values were also recorded at 600 nm to investigate the bacterial concentration. After comparing the TC degradation efficiency and growth of each bacterial consortium, the best TC-degrading bacterial consortium was selected for subsequent experiments.

### 2.4. Lab-Scale Soil Remediation Using the Best TC-Degrading Bacterial Consortium

The lab-scale soil remediation was conducted to evaluate the effectiveness of the TC-degrading bacterial consortium in the soil environment. Soil samples obtained from hoggery were sieved through a 0.3-mm-mesh screen and premixed uniformly, and then separately filled into cylindrical containers (500 g soil per group and the height of the soil layer is 5 cm) to simulate two different soil environments. In addition to soil properties, temperature is another factor that needs to be considered and which may also affect the bioactivity of TC-degrading bacteria. Hence, soil samples were placed in incubators of 5 °C and 30 °C to investigate the consequent effects. Afterwards, the bacterial suspensions were added to the soils, once every 5 days for 1 month, at a concentration of 10^7^ cells/g, and each soil was mixed thoroughly after the addition of bacteria. Moreover, Hemacytometry showed that the bacterial concentration of the bacterial consortium reached 5 × 10^8^ cells/mL after shake-flask cultivation for 24 h, so the addition volume was determined to be 10 mL. Soils added with the same amount of inactivated bacterial suspensions were set as control groups, and all experiments were triplicated. During the microbial remediation, concentration of TC was detected via HPLC. Soil samples were collected to analyze the variations in bacterial communities, TRGs, MGEs, and changes in soil properties were also recorded (Table S1).

### 2.5. Quantification of TC and Its Sample Pretreatment

TC concentration in soil was detected by HPLC (Hitachi 8DD-0801) with a C18 reversed-phase column (Agilent TC-C18(2) 150 mm × 4.6 mm). Instrument parameter setting, operation, and pretreatment of liquid samples were performed according to the previous study [14]. Besides, soil samples were treated as follows: In a 10 mL centrifuge tube, 5 mL of methanol, and EDTA-McIlvaine (1/1, *v*/*v*) buffer were added to 2 g of soil samples, then oscillated at 25 °C for 10 min, ultrasonicated for 10 min, and centrifuged at 4500 rpm/min for 10 min. The supernatant was collected and the same procedures were performed for two more times to remnants in the tube. The total extracts were treated with a rotary evaporator in a dark environment until the volume remained unchanged. Solid-phase extraction (SPE) was performed to purify the soil extracts with SPE vacuum manifolds. Hydrophilic–lipophilic balance (HLB) cartridges (6 mL per 150 mg, Copure, China) were preconditioned by washing sequentially with 6 mL of methanol and 6 mL of EDTA-McIlvaine. The obtained supernatant was passed through the HLB cartridges. After loading and isolation, the cartridges were washed with 6 mL ultrapure water and dried for 10 min in the vacuum oven. The retained TC residuals were eluted with 3 mL of methanol, and the eluent was dried with nitrogen gas in a water bath at 40 °C. The obtained solid samples were dissolved with 1 mL of methanol and water solution (3/2, *v*/*v*), filtered through a 0.22 µm membrane filter and stored in a 1.5 mL amber vials at −20 °C prior to HPLC analysis.

### 2.6. DNA Extraction of Soil Samples

Soil samples were collected for DNA extraction, including original soils and soils in which the microbial remediation had been carried out for 65 days, and DNA extraction was performed immediately after the sample were collected. Genomic DNA was extracted using the DNeasy^®^ PowerSoil^®^ Kit (50) (QIAGEN, Dusseldorf, Germany) according to the manufacturer’s instructions, and DNA concentration was measured by spectrophotometric analysis on a NanoDrop ND-1000 (Nanodrop, Waltham, MA, USA), The DNA samples were then stored at −20 °C for further analysis.

### 2.7. 16S rRNA Gene Amplification, Sequencing, and Data Processing

To get an insight into the shifts in bacterial communities, the V4-V5 region of the bacterial 16S rRNA gene was amplified with primer 515F (5′-GTGCCAGCMGCCGCGG-3′), and primer 907R (5′-CCGTCAATTCMTTTRAGTTT-3′) [15]. The PCR protocol was prepared and conducted according to the previous study [16]. The subsequent high-throughput sequencing was operated by Majorbio Bio-Pharm Technology Co., Ltd., (Shanghai, China) on the Illumina Miseq PE300 platform (Illumina, San Diego, CA, USA).

All the obtained raw sequence reads were submitted to the NCBI database (Accession number provided by the NCBI database: SRR10098497–SRR10098508) and filtered in accordance with the criteria. Operational taxonomic units (OTUs) were clustered at the threshold of 97% using Usearch 7.1. The microbial classification information was generated by RDP Classifier at a confidence threshold of 70% based on the Bayesian algorithm. Alpha diversity indices including Chao1, Shannon, ACE, and Simpson index were calculated by Mothur 1.30.1. Composition and variation of bacterial communities were visualized via Rstudio Version 3.5.0 [17].

### 2.8. Quantification of Tetracycline Resistance Genes (TRGs) and Mobile Gene Elements (MGEs) Using High-Throughput qPCR (HT-qPCR)

In order to figure out whether the introduction of the TC-degrading bacterial consortium would cause the dissemination of TRGs, high-throughput qPCR (HT-qPCR) was employed to quantify the abundance of TRGs. This study selected 21 TRGs and 5 MGEs, which are frequently detected in soil, sludge, and manure, and a total of 37 validated primers (Table S2) were designed according to the previous study [18]. The HT-qPCR analysis was performed using Applied Biosystems ViiATM7 Real-Time PCR System (Wcgene Biotechnology, Shanghai). The qPCR mixture (10 μL) consisted of 5 μL TB GreenTM Premix Ex TaqTM II (Tli RNaseH Plus) (2×), 0.4 μL of each primer, 3 μL ddH_2_O, 0.2 μL ROX Reference Dye (50×), and 1 μL DNA template. The procedures of PCR protocol were as follows: Initial denaturation was performed at 95 °C for 30 s, followed by 40 cycles of reaction including 95 °C for 30 s and 60 °C for 30 s. For each primer set, amplification was performed in triplicate including a non-template control. The specificity of amplification was determined according to the melting curve generated, ranging from 60 °C to 95 °C.

Data of qPCR were optimized using SmartChip qPCR software (v2.7.0.1) according to the previous study [19]. A threshold cycle (Ct) of 40 was used as the detection limit. The relative abundance of TRGs and MGEs were calculated according to equations listed below the paragraph. Ct (TRG) and Ct (16S rRNA) represent the threshold cycles of specific TRG and 16SrRNA gene, respectively. Excel 2016 (Microsoft, Redmond, Washington, DC, USA) was used to process the obtained data, and several diagrams were plotted in Origin Pro 9.1 software (OriginLab, Northampton, MA, USA) to indicate the variations in TRGs and MGEs. Network analysis was performed in Gephi Version 0.9.2 to visualize the correlation between TRGs, MGEs, and bacteria. Data used for plotting was generated by SPSS Statistics V24.0 (IBM, Armonk, NY, USA).

ΔCt = Ct (TRG) − Ct (16SrRNA),(1)

F = 2^−Δ*C*t^, (2)

## 3. Results and Discussion

### 3.1. Strain Isolation and Construction of TC-Degrading Bacterial Consortium

Soil suspensions of the two samples were acclimated in LB medium with increasing TC concentration. When the TC concentration reached 180 mg/L, the bacterial solution was diluted and daubed on Petri dishes made of mineral medium with 50 mg/L TC for further purification. Eventually, two bacterial strains were obtained, their growth curves in the mineral medium with 50 mg/L TC are shown in Figure S1, which indicated that both strains were able to thrive using TC as their nutrient substance despite that the lag phase was long and the bacterial concentration was low. In addition, *A. faecalis* S-1 exhibited better growth in comparison to *A. faecalis* S-2. Table S3 shows that the two strains differ in nitrate reduction and urease activity. After submitting their 16s rRNA sequences to the NCBI database, the BLAST result indicated that both strains have the highest similarity to one species: *Alcaligenes faecalis*, so the two strains were named *A. faecalis* S-1 (*A. faecalis* S-1 shares 99.93% similarity with *A. faecalis* strain 309-5, *e* value < 1 × 10^−179^) and *A. faecalis* S-2 (*A. faecalis* S-2 shares 99.93% similarity with *A. faecalis* strain FC2960, *e* value < 1 × 10^−179^), respectively. *A. faecalis* was investigated in many studies, and it was reported that strains belonging to this species exhibited excellent ability in biodegrading organic pollutants [20,21,22], including aromatic and heterocyclic compounds. More specifically, *A. faecalis* is capable of secreting enzymes associated with O-dealkylation, cleavage of aromatic rings and C-N bond, and other oxidative reactions [20,21,22], which may account for the biodegradation of TC since such structures exist in the TC molecule.

**Figure 1:**
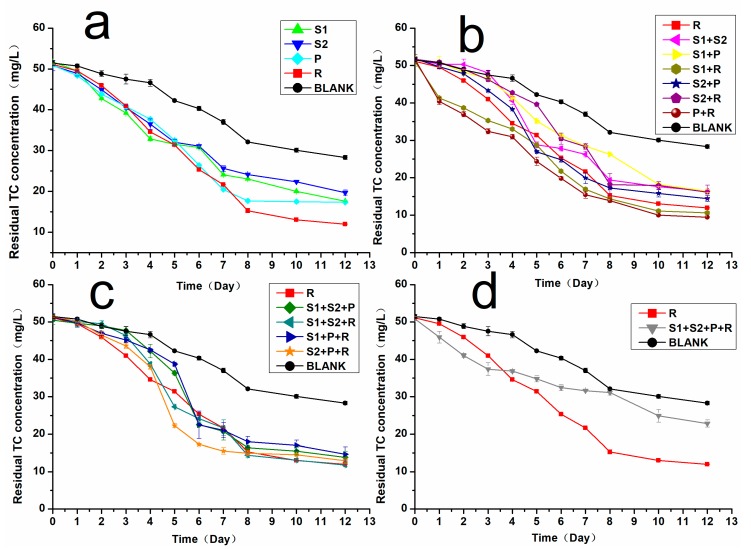
Residual tetracycline (TC) concentration in the lysogeny broth (LB) medium: (**a**) TC degradation of each single strain, (**b**–**d**) Comparision between the best strain (*Raoultella* sp. XY-1) and the bacterial consortia. S1: *A. faecalis* S-1; S2: *A. faecalis* S-2; P: *Pandoraea* sp. XY-2; R: *Raoultella* sp. XY-1. BLANK stands for control groups without adding bacteria.

TC degradation efficiency of each strain and the constructed bacterial consortium were evaluated, respectively, and results are shown in Figure 1. Overall, the variation trend of most curves was somewhat similar, and only a slight decrease of TC concentration occurred in the early stage, which was mainly due to the low number of bacteria, as the growth of bacteria was inhibited in the lag phase. However, strain combinations of S1&R, P&R, and S1&S2&P&R showed a rapid TC-degrading rate since the beginning of the experiment, implying that there may exist certain biochemical synergy effects between the bacteria involved, which enhanced the bacterial activity and TC degradation. As the logarithmic growth phase began, TC concentration dropped more rapidly, indicating that the biodegradation of TC would be enhanced once the bacteria reached certain amounts. However, the rapid decline of TC concentration only lasted for 3 days on curve S1&S2&P&R, while the fast downward trend of other curves lasted until day 8, despite little fluctuations. S1&S2&P&R was the only bacterial consortium containing four strains, and the complexity of the system may result in certain depression among bacteria, thus affecting the TC degradation efficiency. As the experiment proceeded, the decline of TC concentration became slower on all curves, which was mainly due to the death of bacteria and decrease in the number of bacteria, and the final TC concentration was recorded on day 12. Results indicated that TC concentration in control groups decreased by nearly half of its initial amount (44.99%) owing to hydrolysis [23] and *Raoultella* sp. XY-1 biodegraded 76.63% of TC, which was the best among the four strains. As for the constructed bacterial consortium, S1&R (79.15%), P&R (81.72%), and S1&S2&R (77.09%) showed better TC degradation, and P&R (81.72%) achieved the best degradation efficiency of TC in all groups, implying that the biochemical synergy effect of certain bacterial combinations could, to some extent, enhance the biodegradation of TC. Many studies reported such instances of enhancing organic pollutants removal through microbial cooperation [24,25]. The results of t-test (Table S4) also indicated that the TC-degrading efficiency of P&R was significantly improved in comparison to that of *Raoultella* sp. XY-1 which is the best TC-degrading strain. Nevertheless, the TC degradation efficiency of most strain combinations did not improve or even decrease, which may be caused by growth competition between strains and inhibition effect of specific metabolites. As illustrated in Figure S2, the growth of different bacterial consortium varies. For instance, the lag phase of most groups was around 4 h, while the bacterial concentration of S1&S2&P&R remained unchanged until 10 h, which may account for its lower TC degradation efficiency. Additionally, for other bacterial consortia with unimproved or decreased TC degradation efficiency, the maximum bacterial concentration they reached was also relatively lower. Hence, it may be concluded that random combinations of TC-degrading bacteria would not necessarily enhance the biodegradation of TC, and the TC degradation efficiency may decline when the bacterial consortium get complex since the living conditions could be too competitive for different bacteria to coexist, let alone to biodegrade hazardous chemicals. Fortunately, in this study, the bacterial consortium containing *Pandoraea* sp. XY-2 and *Raoultella* sp. XY-1 was found to exhibit better growth and improved TC degradation efficiency, and the lab-scale soil remediation was subsequently carried out to test its effectiveness in the soil environment.

### 3.2. Effectiveness of the TC-Degrading Bacterial Consortium in the Soil Environment

**Figure 2:**
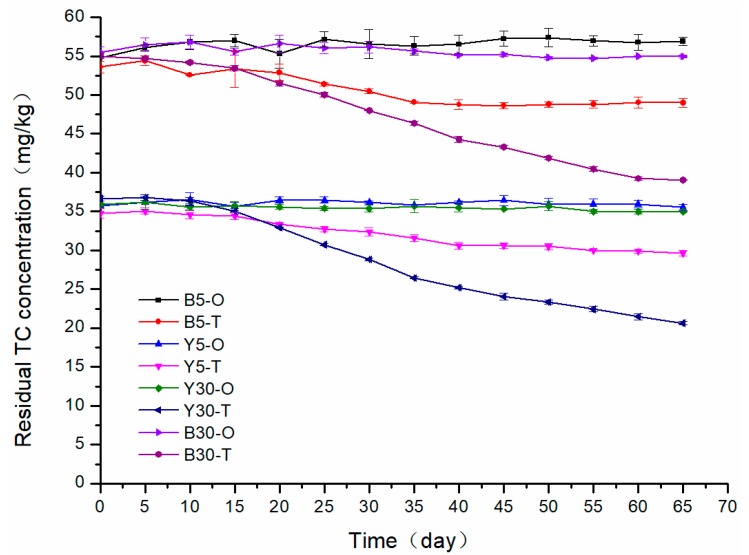
Residual TC concentration recorded in the soil. B and Y indicate the two soil samples. 5 and 30 are the set temperatures in the experiment. T indicates experimental groups, to which the TC-degrading bacterial consortium was introduced, and O indicates control groups, to which the same amount of inactivated bacterial suspensions were added.

Results of TC degradation in the lab-scale soil remediation are shown in Figure 2. TC degradation was not observed in all control groups (B5-O, Y5-O, B30-O, and Y30-O) regardless of temperature and soil type, while TC concentration decreased in soils in which the TC-degrading bacterial consortium was introduced despite that the TC degradation efficiency was much better at 30 °C than 5 °C (B5-T: 8.57%, B30-T: 28.95%, Y5-T: 14.80%, Y30-T: 43.72%). Hence, it was concluded that the TC-degrading bacterial consortium was effective in biodegrading TC in soil, but the effectiveness was affected by soil type and temperature, and to some extent, the influence of temperature seemed to be greater. Though the TC degradation efficiency of the two soils varied due to the different initial TC concentration and soil properties, they both exceeded 25% at 30 °C. However, at 5 °C, the TC degradation efficiency was lower than 15% for both soils. Therefore, practical application of the TC-degrading bacterial consortium should be carried out in summer so as to achieve better results, since bacteria tend to show higher bioactivity in high temperature, and the molecular reaction also increases with an increase in temperature, which may promote the desorption of TC from soil, making the target pollutant more bioavailable [26,27].

Though the TC degradation efficiency of the bacterial consortium was less affected by soil properties, the final results of sample B and sample Y were different. Because the bioactivity of bacteria was severely inhibited at 5 °C, the TC degradation was similarly low in both soils, while in experiments conducted at 30 °C, the TC-degrading efficiency varied significantly between sample B and sample Y, 28.95% for B30-T and 43.72% for Y30-T. As shown in Table S1, the initial TC concentration of sample B (about 55 mg/kg) was higher than that of sample Y (about 35 mg/kg), and the high TC concentration seemed to inhibit the effectiveness of the bacterial consortium, which may be due to the low bioactivity of bacteria under huge TC pressure, or the low bioavailability of TC caused by more adsorption of TC to soil. Thus, better TC degradation efficiency was recorded in sample Y which had lower initial TC concentration. Besides, the bioactivity of bacteria and the bioavailability of TC were also related to soil properties, which may be attributed to the differences in TC degradation in the two soils. For instance, soil pH was reported to be the main determinant affecting the adsorption of TC to the soil, and the TC molecule bonded more strongly to soil particles under acidic conditions [28]. In this study, the initial soil pH of sample B and sample Y were approximately 6 and 4, respectively, indicating that there may exist stronger adsorption between TC and soil in sample Y30. However, a distinct increase in soil pH was recorded in experimental groups of sample Y at 30 °C (from about 4 to about 6), indicating that the introduction of the TC-degrading bacterial consortium increased the soil pH from acid to near neutral, which promoted the desorption of TC from soil and made the pollutant more bioavailable [29]. Eventually, TC degradation was enhanced. In addition, soil moisture content (SMC) was another factor to consider, because high SMC could increase the oxygen content in soil and support certain aerobic degradation pathways of bacteria. However, both soil samples have high SMC values at the initial stage of the microbial remediation (B30: about 30%, Y30: about 27%), and the SMC values similarly declined as the experiment proceeded, so it seemed untenable to attribute the different TC degradation efficiency in the two soils to SMC values. Moreover, soil organic matter (SOM) could interact with the TC pollutant in the soil through cation exchange, which may reduce the bioavailability of TC. As shown in Table S1, the initial SOM values of sample Y were slightly lower than that of sample B, which may contribute to the better TC degradation recorded in sample Y. However, the interaction between SOM and TC was significantly affected by soil pH [30], so the SOM value barely contributed to the differences in TC degradation recorded in the two soils. Besides, the amount of total carbon (TOC) was found to be negatively correlated with the sorption of TC to the soil, implying that the TC-degrading bacterial consortium may achieve better TC degradation in soil with high TOC values. Better results were recorded in sample Y which had relatively lower TOC values, thus it can be seen that TOC was also a less significant factor, affecting the effectiveness of the bacterial consortium in soil. Additionally, metal ions were reported to have great impacts on the biodegradation of TC in soil [1]. The formation of chelate complexes between TC and metal ions was the primary issue, and Cu^2+^, Ca^2+^, Mg^2+^, and Na^2+^ were the main divalent cations involved. Table S1 showed that there existed more Cu^2+^, Ca^2+^, and Mg^2+^ in sample Y, implying that TC may be less bioavailable in sample Y, but the TC degradation recorded in sample Y turned out to be better than sample B, which could also be attributed to soil pH since with the increase of soil pH, the sorption between TC and most metal ions would gradually weaken. Soil pH of sample Y increased from acidic to near neutral after 65 days of bioremediation, and this may result in the better TC degradation efficiency. To conclude, at 30 °C, the TC degradation efficiency of the bacterial consortium would be significantly affected by initial TC concentration, because high TC pressure could inhibit the bioactivity of bacteria and reduce the bioavailability of TC. Moreover, soil pH is the main factor determining the bioavailability of TC in soil, and the introduction of the bacterial consortium could increase the soil pH to near neutral, which promoted the desorption of TC from soil and thus enhanced the TC-degrading efficiency. Besides, the TC-degrading bacterial consortium seemed to function well regardless of the soil pH, and even achieved better TC degradation in acid conditions.

### 3.3. Shifts of Bacterial Communities during the Lab-Scale Soil Remediation

Since the microbial remediation carried out at 30 °C achieved better TC degradation, it was worthwhile to investigate the shifts of soil bacterial communities before and after the introduction of the TC-degrading bacterial consortium. After the high-throughput sequencing analysis, a total of 460,485 high-quality sequences were obtained from twelve soil samples, which generated 3455 OTUs at a similarity level of 97.00%. Detail information of each soil sample was shown in Table S5, including sequence number, OTUs number, and alpha diversity indices. Though the three duplications of each soil sample were collected from the same site and barely had any differences in soil properties, they differed in bacterial communities, and not only just in bacterial diversity and richness but also in bacterial composition, as demonstrated by the PCoA analysis (Figure S3). From our perspective, the heterogeneity of the soils led to the differences in their bacterial communities, since the soils were mixed with small particles and decaying leaves when collected. Thus, different parts of the soil may contain different microbiota. The data obtained from each duplication were combined, and the redundant OTUs were removed. The OTUs number generated from each soil was as follows: 2606 for B30-O, 2325 for B30-T, 2625 for Y30-O, and 1860 for Y30-T. It indicated that the OTUs number of both soils decreased after the bioremediation, which could be caused by the introduction of the bacterial consortium, since the foreign bacteria may outcompete the indigenous ones and lead to the decline in bacterial richness or could have resulted from the decrease in TC concentration because the previous dominant species were more accustomed to high TC pressure, and once the TC concentration decreased, those bacteria could no longer maintain their competitive edge over the others so the bacterial diversity declined. In addition, changes in soil properties should be taken into consideration, such as decrease in SMC due to evaporation of water, an increase in soil pH and TOC after long-term bioremediation, etc. All these potentially affected the soil bacterial communities. The Venn diagram was made to illustrate the specific and shared OTUs of different soils. As is shown in Figure S4, B30-O and B30-T had 1971 shared OTUs, while Y30-O and Y30-T had 1413 shared OTUs, which indicated that the microbial remediation generated great impacts on the soil bacterial communities, especially on sample Y. Moreover, sample B and sample Y had 2278 shared OTUs before the introduction of the TC-degrading bacterial consortium, suggesting that the indigenous bacterial communities of the two soils were similar despite the initial TC concentration and soil properties being different. After the bioremediation, the shared OTUs of the two soils dropped to 1135, which may indicate that the microbial remediation had different effects on the bacterial communities of the two soils, and the abundance of different bacterial species exhibited different variation trends due to their distinct responses to the introduction of the foreign bacteria. 

**Figure 3:**
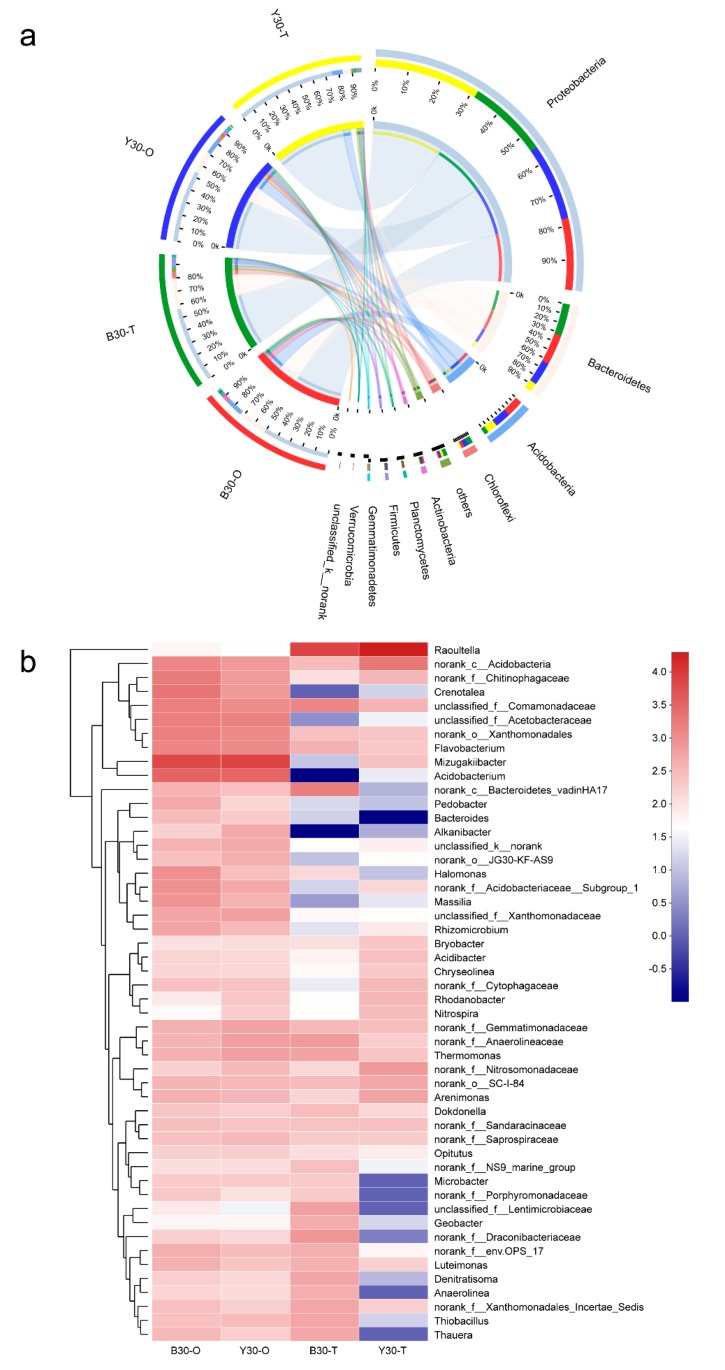
(**a**) Circos diagram showing the relative abundance of the bacterial communities at the phylum level, (**b**) Heatmap showing the relative abundance of bacterial communities at the genus level. B30-O and Y30-O stand for original soil samples, while B30-T and Y30-T stand for soil samples collected on day 65.

The relative abundance of soil bacterial communities at the phylum level is shown in Figure 3a. It indicated that *Proteobacteria*, *Bacteroidetes*, *Acidobacteria*, and *Chloroflexi* were the four dominant phyla in soil, and *Proteobacteria* had the highest abundance in all groups, 55% for Y30-O, 77% for Y30-T, 52% for B30-O, and 58% for B30-T. Previous studies reported that bacteria belonging to *Proteobacteria*, such as α-, β-, and γ- *Proteobacteria*, exhibited high resistance to antibiotics and predominated in bioreactors processing TC and other antibiotics [31], which may account for the large abundance of *Proteobacteria* in the soils. Moreover, the relative abundance of *Proteobacteria* showed a significant increase in both soils after 65 days of bioremediation. Given the fact that the TC-degrading bacterial consortium was comprised of two strains both belonging to *Proteobacteria*, an assumption was made that the introduction of the bacterial consortium caused the increase in *Proteobacteria*. This may imply that the bacterial consortium successfully proliferated in the soils. 

To prove the speculation, a more in-depth analysis of the bacterial community at the genus level was conducted. A heatmap (Figure 3b) of the 50 most abundant bacterial genus in soil was plotted, which showed that the relative abundance of *Raoultella* sharply increased in both soils after 65 days of microbial remediation (sample Y: from 0.19% to 54.64%, sample B: from 0.13% to 22.40%), while *Pandoraea* was undetectable. Thus it can be seen that the bacterial consortium could not function cooperatively to biodegrade TC in the soil, since *Pandoraea* sp. XY-2 failed to proliferate. This could be attributed to two reasons: on the one hand, the soil environment was much more complicated than the shake-flask cultivation and was not suitable for the growth of *Pandoraea* sp. XY-2. On the other hand, *Pandoraea* sp. XY-2 was isolated from other soil samples, and it may be difficult for foreign bacteria to survive in different soil environments. However, fortunately, *Raoultella* sp. XY-1 successfully proliferated after being introduced into the soil, and considerable TC degradation was achieved. In addition, the relative abundance of some bacteria declined after the microbial remediation, for instance, these bacterial genera: *Thauera*, *Draconibacteriaceae*, *Porphyromonadaceae*, *Massilia*, *Microbactor*, *Anaerolinea*, *Bacterioides*, *Crenotalea*, *Cytophagaceae*, *Rhizomicrobium*, *Alkanibactor*, *Pedobactor*, *Acidobacterium*, *Mizugakiibacter*, *Acetobacteraceae*, and *Chitinophagaceae*. Some of them are N-cycling-related bacteria, which have been shown to play important roles in nitrogen removal (*Thauera*, *Anaerolinea*, *Mizugakiibacter*, *Chitinophagaceae*) and nitrogen fixation (*Rhizomicrobium*, *Acidobacterium*, *Acetobacteraceae*). Some of them are involved in carbohydrate metabolism (*Porphyromonadaceae*, *Chitinophagaceae*) and biodegradation of organic pollutants (*Massilia*, *Anaerolinea*, *Acetobacteraceae*, *Acidobacterium*, *Rhizomicrobium*, *Cytophagaceae*), and some are beneficial bacteria related to soil biochemical reactions and plant growth (*Cytophagaceae*, *Massilia*, *Acidobacterium*, *Mizugakiibacter*). In summary, the introduction of the TC-degrading bacterial consortium to soil may generate negative impacts on soil functional microbiota.

### 3.4. Variations in TRGs and MGEs during the Lab-Scale Soil Remediation

TRGs are emerging contaminants concurrent with TC, potentially threatening the environment. For instance, TRGs are genes that replicate themselves, which means they are difficult to eliminate even if the TC pressure is no longer existing, and once the proliferation starts, it is hard to maintain control. In addition, TRGs could be transferred among microbes via horizontal gene transfer, which is likely to confer resistance to human pathogens and pose a risk to human health [32]. The currently known TRGs mainly involve three specific mechanisms: efflux pump of TC, target modification to ribosomal protection protein, and inactivation via enzymatic alteration [33]. These genes may empower microbes to survive under the TC pressure via MGE-mediated horizontal gene transfer. Hence, the content of TRGs and MGEs should be considered as a part of environmental safety assessment.

As illustrated in Table 1, the detection rates of TRGs and MGEs belonging to different mechanisms were different between the four groups (B30-O, Y30-O, B30-T, and Y30-Y). As for the overall detection rate of TRGs in the original soils, sample B was higher than sample Y, which could be attributed to the higher TC concentration in soil sample B, since the higher selective pressure of antibiotics may proliferate the TRGs hosts, thus leading to more frequent incidence of TRGs. A previous study reported that high detection rate of ARGs indicated more existence of ARG hosts [5], hence there may exist more TRGs-carrying microbes in soil sample B. In addition, differences in soil properties may also lead to different detection rates of TRGs. For instance, TRGs were found to be more prevalent in wet soils than in dry soils [7], which was in accordance with the obtained result, as soil sample B had a higher SMC than soil sample Y. Additionally, soil pH, SOM, and TOC of soil sample B were relatively higher (Table S1), which may contribute to more frequent occurrence of TRGs. The overall detection rate of MGEs in original soils (B30-O and Y30-O) was similar between the two samples. However, the overall detection rates of both TRGs and MGEs surprisingly declined after the microbial remediation, which may indicate that the introduction of the TC-degrading bacterial consortium was less likely to cause proliferation of TRGs and MGEs. 

**Figure 4:**
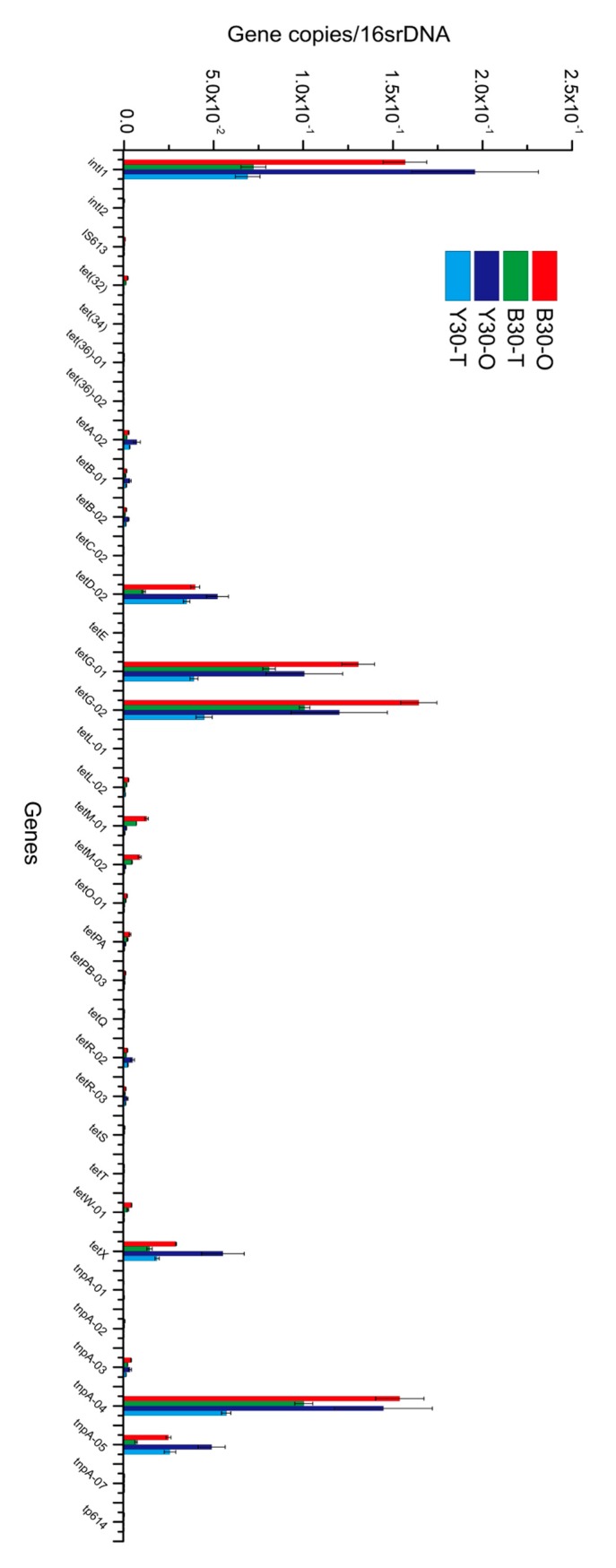
Relative abundance of tetracycline resistance genes (TRGs) and mobile gene elements (MGEs) before and after the microbial remediation. B30-O and Y30-O stand for original soil samples, whiles B30-T and Y30-T stand for soil samples collected on day 65.

To further investigate the detail information, the relative abundance of TRGs and MGEs was quantified. As illustrated in Figure 4, TRGs including *tetD*, *tetG*, and *tetX* were verified to have higher relative abundance in soil, which was in accordance with the previous studies reporting that efflux pump genes of TC (*tetA*, *tetB*, *tetC*, *tetD*, *tetE*, and *tetG*) were ubiquitous in soils [34,35]. Moreover, the relative abundance of *tetX* was approximately 10^−2^ gene copies/16rDNA, which may indicate that there existed strong biodegradation of TC in the soil since *tetX* was the only TRGs capable of degrading TC via enzymatic modification [33]. As for MGEs, *intI1*, *tnpA-04*, and *tnpA-05* exhibited higher relative abundance, and *intI1* was proved to be closely associated with the propagation of ARGs [36], suggesting that the integrase-mediated gene transfer was much likely to occur in the soil environment. However, the relative abundance of most TRGs and MGEs decreased after the microbial remediation, so it could be concluded that the application of the TC-degrading bacterial consortium in the soil would not cause the proliferation of TRGs and MGEs. One explanation for this result could be that the relative abundance of the indigenous bacteria gradually decreased after the foreign bacteria became dominant species, and due to the long-term stimulation of TC, a vast majority of the indigenous bacteria could be hosts of TRGs, thus, their reduction led to the downregulation of TRGs and MGEs content. Or perhaps as the microbial remediation proceeded, those bacteria which are more accustomed to higher TC pressure may no longer maintain their competitive edges and died due to the decline in TC concentration, subsequently reducing the relative abundance of TRGs and MGEs. Additionally, changes in soil properties throughout the bioremediation may have affected the indigenous bacteria and eventually led to a decrease of TRGs and MGEs. Hence, it seemed promising to apply the TC-degrading bacterial consortium to practical soil bioremediation, as it would not pose any threats to the environment in terms of TRGs contamination.

### 3.5. Correlations between TRGs, MGEs, and Bacterial Communities

**Figure 5:**
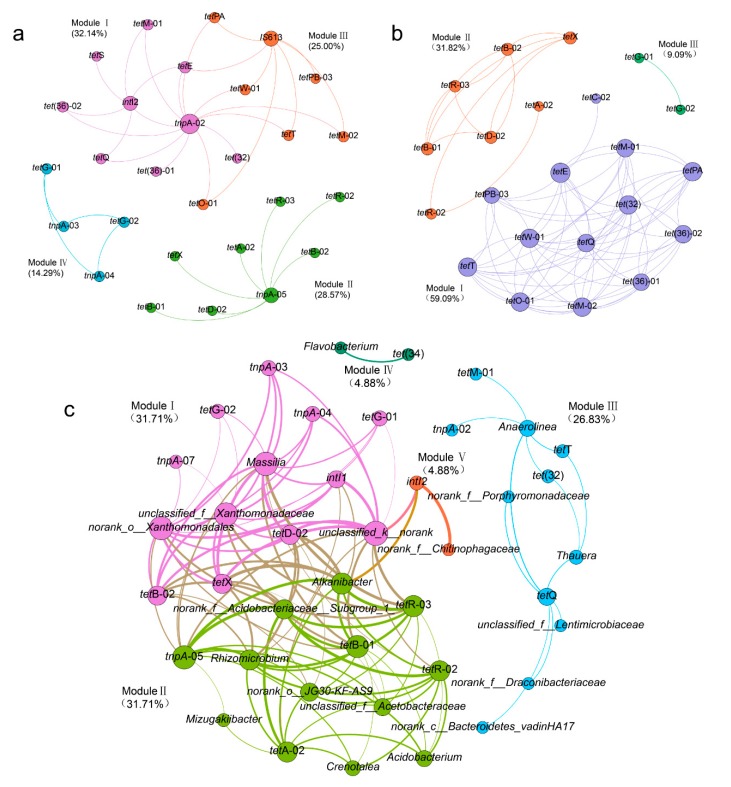
Network analysis: (**a**) predicts the dissemination of specific TRGs facilitated by MGEs, (**b**) reveals the co-occurrence of TRGs, (**c**) identifies the potential hosts of TRGs and MGEs.

Network analysis was performed to predict the MGE-mediated gene transfer of TRGs (Figure 5a), to reveal the co-occurrence of TRGs (Figure 5b) and to identify the potential hosts of TRGs and MGEs (Figure 5c). Data with a statistically significant correlation (*p*-value) were retained for visualization. In Figure 5, the value of Spearman’s rank correlation coefficients (r) was proportional to the thickness of each edge between nodes, the size of node in the network was positively correlated with the number of connections, and the node with the most linkages was considered ‘hub’, which could be recognized as the indicator of its associated genes. The modularity generated by Gephi-0.9.2 indicated the division of the network. Each module accounted for different proportions and the biggest one may be considered as the representative of the association between TRGs, MGEs, and bacterial communities in soil. 

Figure 5a (r > 0.9, *p*-value < 0.01) visualized the correlation between TRGs and MGEs, which revealed the possibility of MGE-mediated gene transfer of TRGs among microbes. Module I (39.29%) and Module II (28.57%) were the two largest modules, suggesting that the transfer of TRGs was mainly dominated by *tnpA*. Hence, an assumption was made that the transposase played a more important role in the horizontal transfer of TRGs compared with integrase. Besides, *tetE*, *tetG*, and *tetM* linked with multiple MGEs, suggesting that they were more likely to spread among microbes. However, fortunately, the HT-qPCR results indicated that the relative abundance of these genes decreased after the microbial remediation, which may imply that the incidence of horizontal gene transfer was not as high as predicted by the network. Moreover, it was believed that the close association between genes may indicate that they are located on the same gene cluster, hence Figure 5b (r > 0.9, *p*-value < 0.01) was made to show the co-occurrence of TRGs. The network was comprised of three modules, and Module I was the largest one, accounting for 59.09% of the whole network. Though it was the most complex module, the relative abundance of the involved TRGs were all lower than 10^-3^ gene copies/16rDNA, implying that they may not be the main cause of TC resistance in the environment. Module II accounts for 31.82% of the network, and the involved *tetX* and *tetD* all had high abundance, which may indicate that the close interaction between the two TRGs could be the main contributor to the TC resistance. Lastly, in order to identify the potential hosts of TRGs and MGEs, 50 most abundant bacterial genera were selected for network construction (Figure 5c, *p*-value < 0.05, Spearman’s r-values ranging from 0.58 to 0.89). Results showed that *Massilia*, *Alkanibacter*, *Rhizomicrobium*, *Xanthomonadales*, *Acidobacteriaceae*, and *Xanthomonadaceae* were associated with more TRGs and MGEs, so they could be considered as possible hosts of TRGs and MGEs. In addition, the abundance of these bacteria gradually declined as the bioremediation proceeded, and so did the relative abundance of most TRGs and MGEs. Hence, the prediction of the network seemed tenable, since microbes were hosts of TRGs and MGEs, and the decrease of hosts would inevitably lead to the decrease of the related genes. Moreover, *Raoultella* was not significantly correlated with any TRGs or MGEs in the network, so it could be concluded that the application of the TC-degrading bacterial consortium would not pose significant threats to the environment.

## 4. Conclusions

In this study, a TC-degrading bacterial consortium containing *Raoultella* sp. XY-1 and *Pandoraea* sp. XY-2 was constructed, which was capable of biodegrading 81.72% TC within 12 days in LB medium. The lab-scale soil remediation further proved that the application of the TC-degrading bacterial consortium in the soil environment was feasible, despite that the effectiveness could be affected by temperature, initial TC concentration, and soil properties. The best TC degradation was recorded in soil sample Y at 30 °C, in which TC concentration decreased by 43.72% within 65 days. High-throughput sequencing analysis revealed that the soil bacterial diversity declined after the microbial remediation, and the soil functional microbiota was affected to a certain extent. Moreover, only *Raoultella* sp. XY-1 successfully proliferated, while *Pandoraea* sp. XY-2 was undetectable after being added to the soil, suggesting that *Raoultella* sp. XY-1 was the main contributor to TC degradation in soil. Lastly, results of HT-qPCR indicated that *tetD*, *tetG*, *tetX*, *intI1*, and *tnpA* had high relative abundance in soil, and most TRGs and MGEs were down-regulated after the bioremediation, which mainly could be attributed to the decline of host bacteria. In addition, as predicted by the network, *Raoultella* was not significantly correlated with any TRGs or MGEs, indicating that no environmental risks would be caused by the application of the TC-degrading bacterial consortium. Hence, this study successfully developed a microbial method for treating TC-contaminated soil.

## Figures and Tables

**Table 1:** Detection rate of TRGs and MGEs belonging to different mechanisms. B30-O and Y30-O stand for original soil samples, whiles B30-T and Y30-T stand for soil samples collected on day 65.

Groups	TRGs			MGEs	
	Cellular Protection	Efflux Pump	Unknown	Transposase	Integrase
B30-O	100%	94.87%	83.33%	95.83%	100%
B30-T	100%	92.31%	100 %	91.67%	100%
Y30-O	90.90%	84.61%	100%	100%	50%
Y30-T	81.82%	76.92%	100 %	100%	50%

## Supplementary Materials

The following are available online at https://www.mdpi.com/2076-2607/8/2/292/s1, Table S1: Physicochemical parameters of soil samples at 30 °C incubator; Table S2: Primers used in this study; Table S3: Physiological and biochemical characteristics of *A. faecalis* S-1 and *A. faecalis* S-2; Table S4: T-test results of the tetracycline degradation efficiency of *Raoultella* sp. XY-1 and P&R; Table S5: Indices of soil bacterial community richness and diversity; Figure S1: Growth curves of *A. faecalis* S-1 and *A. faecalis* S-2 in the mineral medium with 50 mg/L TC; Figure S2: Growth curves of bacteria in the LB medium with 50 mg/L TC; Figure S3: Principal coordinate analysis showing the distribution pattern of the bacterial communities; Figure S4: Venn diagram of the specific and shared OTUs in all soil samples.
