# Supplementary material for: Construction of a Tetracycline Degrading Bacterial Consortium and Its Application Evaluation in Laboratory-Scale Soil Remediation
Source: Microorganisms. 2020 Feb 20;8(2):292. doi: 10.3390/microorganisms8020292 (PMC7074960; doi:10.3390/microorganisms8020292)
Supplement: Supplementary file 1 [file microorganisms-08-00292-s001.pdf]

Supporting materials for

Captions of tables and figures:

Table S1 Physicochemical parameters of soil samples at 30℃ incubator.

Table S2 Primers used in this study.

Table S3 Physiological and biochemical characteristics of A. *faecalis* S-1 and A. *faecalis* S-2.

Table S4 T-test results of the tetracycline degradation efficiency of *Raoultella* sp. XY-1 and P&R

Table S5 Indices of soil bacterial community richness and diversity.

Figure S1. Growth curves of A. *faecalis* S-1 and A. *faecalis* S-2 in the mineral medium with 50 mg/L TC.

Figure S2. Growth curves of bacteria in shake-flask experiments.

Figure S3. Principal coordinate analysis showing the distribution pattern of the bacterial communities.

Figure S4. Venn diagram of the specific and shared OTUs in all soil samples.

Table S1 Physicochemical parameters of soil samples in 30℃ incubator.

Groups	Parameters									
	pH	ITC (mg/kg)	SOM (%)	SMC (%)	TOC (%)	TN (%)	Ca (Wt%)	Cu (Wt%)	Mg (Wt%)	Na (Wt%)
B30-O1	6.17	54.67	6.98	31.13	8.12	0.69	0.25	0.0041	0.37	0.061
B30-O2	6.36	56.36	6.96	31.06	8.12	0.75	0.25	0.0047	0.35	0.067
B30-O3	6.25	55.52	7.00	31.18	8.12	0.73	0.24	0.0048	0.37	0.064
B30-T1	6.07	-	9.20	12.16	9.85	0.89	0.30	0.0060	0.40	0.34
B30-T2	6.14	-	9.13	12.08	9.85	0.90	0.30	0.0056	0.37	0.31
B30-T3	6.11	-	9.19	12.13	9.85	0.87	0.31	0.0065	0.41	0.29
Y30-O1	4.20	36.10	4.42	26.63	1.6	0.21	0.90	0.020	0.47	0.094
Y30-O2	4.19	35.88	4.49	26.75	1.6	0.20	0.89	0.026	0.48	0.098
Y30-O3	4.15	35.67	4.45	26.70	1.6	0.21	0.92	0.020	0.48	0.091
Y30-T1	6.10	-	6.71	8.19	2.34	0.31	0.89	0.023	0.50	0.39
Y30-T2	6.05	-	6.74	8.16	2.34	0.29	0.90	0.026	0.52	0.35
Y30-T3	6.04	-	6.72	8.22	2.34	0.30	0.88	0.021	0.52	0.40

ITC: initial tetracycline concentration; SOM: soil organic matter; SMC: soil moisture content; TOC: total carbon; TN: total nitrogen. B30-O and Y30-O stand for original soil

samples, B30-T and Y30-T stand for soil samples collected on day 65.

Table S2 Primers used in this study.

Gene name	Forward primer	Reverse primer	Classification	Mechanism
16S rRNA	GGGTTGCGCTCGTTGC	ATGGYTGTCGTCAGCTCGTG	na	na
intI-1	CGAACGAGTGGCGGAGGGTG	TACCCGAGAGCTTGGCACCCA	Integron	integrase
intI2	TGCTTTTCCCACCCTTACC	GACGGCTACCCTCTGTTATCTC	Integron	integrase
tnpA-01	CATCATCGGACGGACAGAATT	GTCGGAGATGTGGGTGTAGAAAGT	Transposase	transposase
tnpA-02	GGGCGGGTCGATTGAAA	GTGGGCGGGATCTGCTT	Transposase	transposase
tnpA-03	AATTGATGCGGACGGCTTAA	TCACCAAACTGTTTATGGAGTCGTT	Transposase	transposase
tnpA-04	CCGATCACGGAAAGCTCAAG	GGCTCGCATGACTTCGAATC	Transposase	transposase
tnpA-05	GCCGCACTGTCGATTTTTATC	GCGGGATCTGCCACTTCTT	Transposase	transposase
tnpA-07	GAAACCGATGCTACAATATCCAATTT	CAGCACCGTTTGCAGTGTAAG	Transposase	transposase
Tp614	GGAAATCAACGGCATCCAGTT	CATCCATGCGCTTTTGTCTCT	Transposase	transposase
IS613	AGGTTCGGACTCAATGCAACA	TTCAGCACATACCGCCTTGAT	Transposase	transposase
tet(32)	CCATTACTTCGGACAACGGTAGA	CAATCTCTGTGAGGGCATTTAACA	Tetracycline	cellular protection
tet(34)	CTTAGCGCAAACAGCAATCAGT	CGGTGATACAGCGCGTAAACT	Tetracycline	other/unknown
tet(36)-01	AGAATACTCAGCAGAGGTCAGTTCCT	TGGTAGGTCGATAACCCGAAAAT	Tetracycline	cellular protection
tet(36)-02	TGCAGGAAAGACCTCCATTACAG	CTTTGTCCACACTTCCACGTACTATG	Tetracycline	cellular protection
tetA-02	CTCACCAGCCTGACCTCGAT	CACGTTGTTATAGAAGCCGCATAG	Tetracycline	efflux pump
tetB-01	AGTGCGCTTTGGATGCTGTA	AGCCCCAGTAGCTCCTGTGA	Tetracycline	efflux pump
tetB-02	GCCCAGTGCTGTTGTTGTCAT	TGAAAGCAAACGGCCTAAATACA	Tetracycline	efflux pump
tetC-02	ACTGGTAAGGTAAACGCCATTGTC	ATGCATAAACCAGCCATTGAGTAAG	Tetracycline	efflux pump
tetD-02	TGTCATCGCGCTGGTGATT	CATCCGCTTCCGGGAGAT	Tetracycline	efflux pump
tetE	TTGGCGCTGTATGCAATGAT	CGACGACCTATGCGATCTGA	Tetracycline	efflux pump
tetG-01	TCAACCATTGCCGATTCGA	TGGCCCGGCAATCATG	Tetracycline	efflux pump
tetG-02	CATCAGCGCCGGTCTTATG	CCCCATGTAGCCGAACCA	Tetracycline	efflux pump

Gene name	Forward primer	Reverse primer	Classification	Mechanism
tetL-01	AGCCCGATTTATTCAAGGAATTG	CAAATGCTTTCCCCCTGTTCT	Tetracycline	efflux pump
tetL-02	ATGGTTGTAGTTGCGCGCTATAT	ATCGCTGGACCGACTCCTT	Tetracycline	efflux pump
tetM-01	CATCATAGACACGCCAGGACATAT	CGCCATCTTTTGCAGAAATCA	Tetracycline	cellular protection
tetM-02	TAATATTGGAGTTTTAGCTCATGTTGATG	CCTCTCTGACGTTCTAAAAGCGTATTAT	Tetracycline	cellular protection
tetO-01	ATGTGGATACTACAACGCATGAGATT	TGCCTCCACATGATATTTTTCCT	Tetracycline	cellular protection
tetPA	AGTTGCAGATGTGTATAGTCGTAAACTATCTATT	TGCTACAAGTACGAAAACAAAACTAGAA	Tetracycline	efflux pump
tetPB-03	TGGGCGACAGTAGGCTTAGAA	TGACCCTACTGAAACATTAGAAATATACCT	Tetracycline	cellular protection
tetQ	CGCCTCAGAAGTAAGTTCATACACTAAG	TCGTTCATGCGGATATTATCAGAAT	Tetracycline	cellular protection
tetS	TTAAGGACAAACTTTCTGACGACATC	TGTCTCCCATTGTTCTGGTTCA	Tetracycline	cellular protection
tetT	CCATATAGAGGTTCCACCAAATCC	TGACCCTATTGGTAGTGGTTCTATTG	Tetracycline	cellular protection
tetR-02	CGCGATAGACGCCTTCGA	TCCTGACAACGAGCCTCCTT	Tetracycline	efflux pump
tetR-03	CGCGATGGAGCAAAAGTACAT	AGTGAAAAACCTTGTTGGCATAAAA	Tetracycline	efflux pump
tetW-01	ATGAACATTCCCACCGTTATCTTT	ATATCGGCGGAGAGCTTATCC	Tetracycline	cellular protection
tetX	AAATTTGTTACCGACACGGAAGTT	CATAGCTGAAAAAATCCAGGACAGTT	Tetracycline	other/unknown

Table S3 Physiological and biochemical characteristics of A. *faecalis* S-1 and A. *faecalis* S-2.

	A. faecalis S-1	*A. faecalis* S-2
Gram staining	-	-
Catalase	+	+
H_2S production	-	-
Gelatinase	+	+
Indole production	-	-
Glucose oxidation	-	-
Mannitol oxidation	-	-
Sorbitol oxidation	-	-
Saccharose oxidation	-	-
Nitrate reduction	-	+
Urease	+	-
Citrate utilization	-	-

Table S4 T-test results of the tetracycline degradation efficiency of *Raoultella* sp. XY-1 and P&R

Paired Samples Statistics

		Mean	N	Std. Deviation	Std. Error Mean
Pair 1	*Raoultella* sp. XY-1	.605748277	11	.2859941610	.0862304840
	P&R	.502032382	11	.2655104110	.0800544011

Paired Samples Correlations

		N	Correlation	Sig.
Pair 1	*Raoultella* sp. XY-1 and P&R	11	.975	.000

Paired Samples Test

		Paired Differences					t	df	Sig. (2-tailed)
		Mean	Std. Deviation	Std. Error Mean	95% Confidence Interval of the Difference				
					Lower	Upper			
Pair 1	*Raoultella* sp. XY-1 -P&R VAR00001	.1037158950	.0644729374	.0194393221	.0604023865	.1470294040	5.335	10	.000

Table S5 Indices of soil bacterial community richness and diversity.

Samples	No. of Sequences	OTUs	Chao 1	Ace	Shannon	Simpson	Coverage
B30-O1	38088	1502	1712.726	1718.129	3.885821	0.171023	0.984361
B30-O2	63002	669	1689.65	1672.525	3.278787	0.244169	0.991197
B30-O3	32588	1989	2227.659	2207.8	5.940959	0.00735	0.987827
B30-T1	38164	1911	2169.637	2232.485	5.323721	0.032242	0.980816
B30-T2	27314	1682	2147.487	2172.489	5.297233	0.036417	0.985208
B30-T3	32585	1732	447.3333	497.9402	2.635249	0.182368	0.997535
Y30-O1	40978	279	2330.004	2320.722	5.813801	0.011814	0.988573
Y30-O2	36803	1835	2323.447	2365.471	5.377903	0.036664	0.986715
Y30-O3	41918	1906	1051.763	1382.523	3.361916	0.111696	0.995667
Y30-T1	26281	1328	1881.782	1902.021	4.187993	0.144345	0.990356
Y30-T2	40958	1579	2401.783	2394.934	6.082472	0.00644	0.984841
Y30-T3	41806	1357	1886.55	1900.988	4.988859	0.025033	0.98892

B30-O and Y30-O stand for original soil samples, and B30-T and Y30-T stand for soil samples collected on day 65.

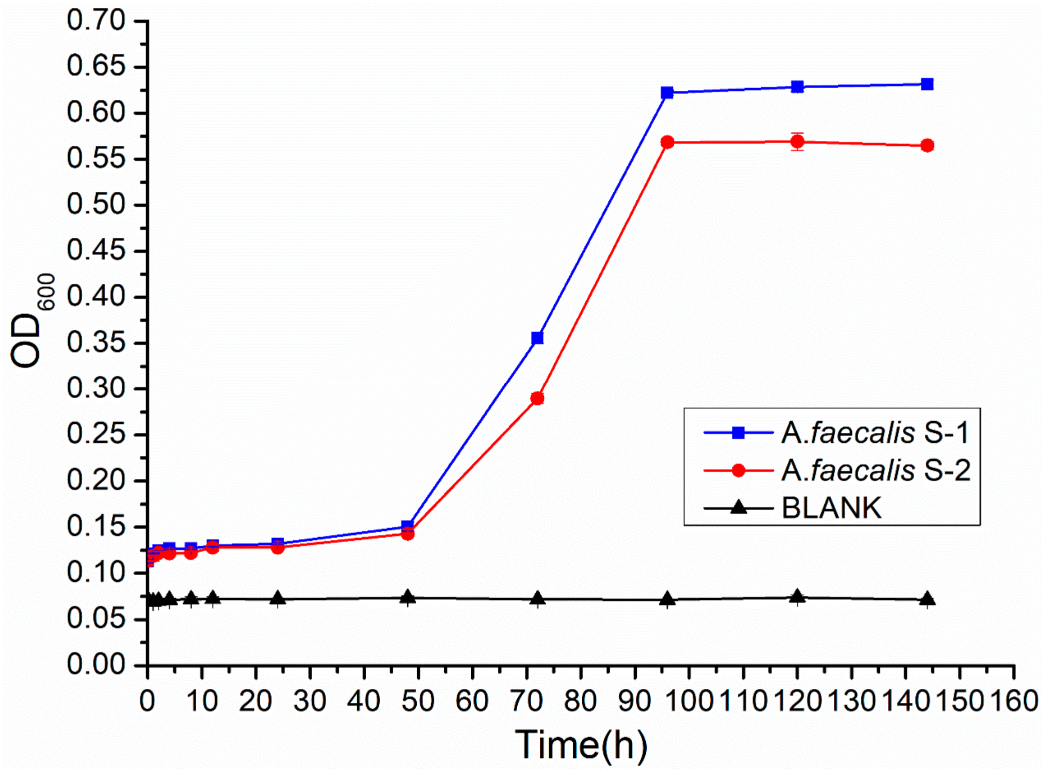

Figure S1. Growth curves of *A. faecalis* S-1 and *A. faecalis* S-2 in the mineral medium with 50 mg/L TC.

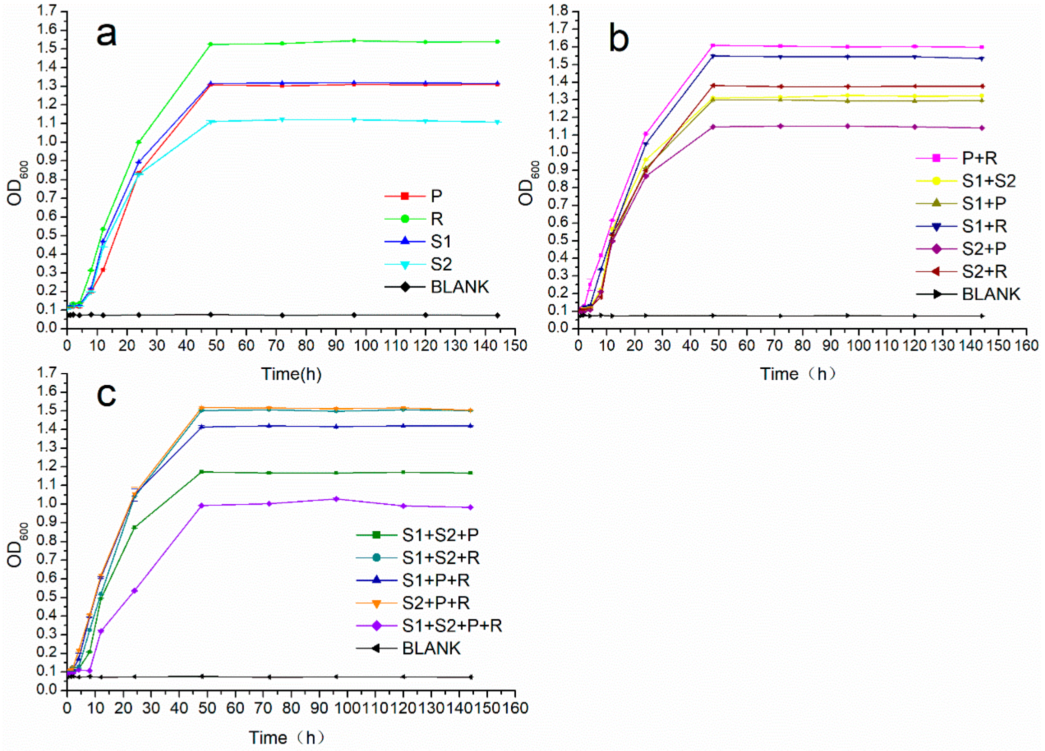

Figure S2. Growth curves of bacteria in the LB medium with 50 mg/L TC: (a) growth curves of the 4 strains, (b) and (c) growth curves of all constructed bacterial consortia. S1: *A. faecalis* S-1; S2: *A. faecalis* S-2; P: *Pandoraea* sp. XY-2; R: *Raoultella* sp. XY-1.

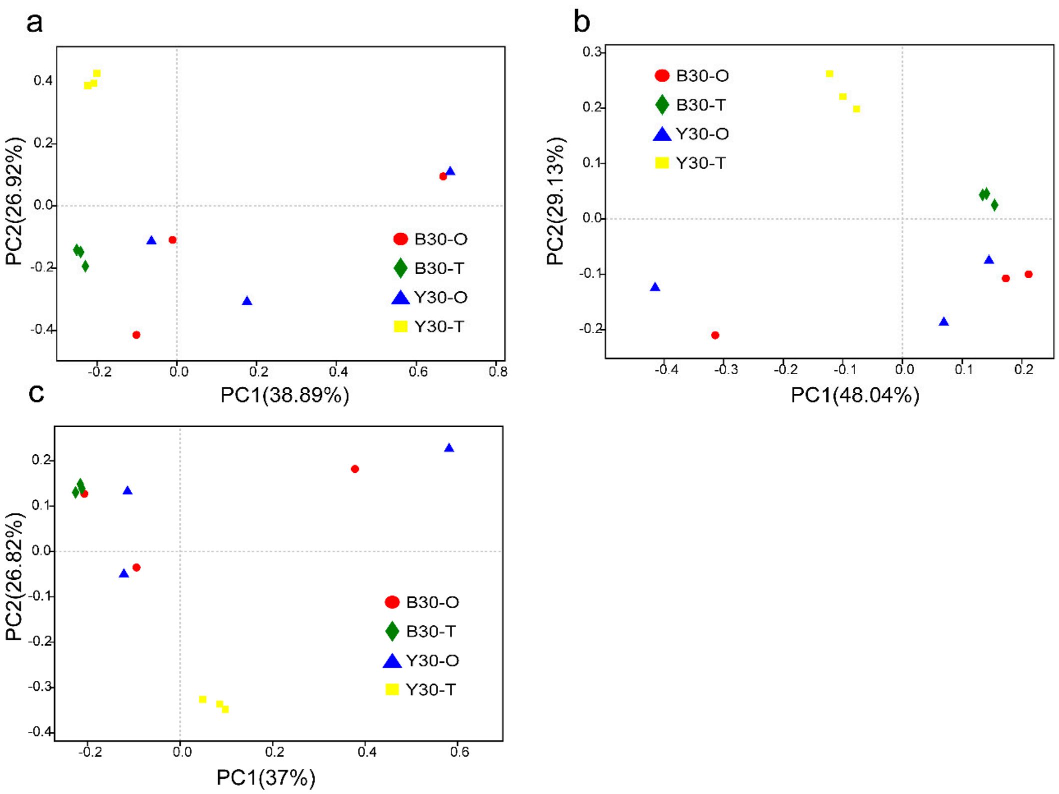

Figure S3. Principal coordinate analysis showing the distribution pattern of the bacterial communities: (a) is based on Bray-Curtis distance; (b) is based on weighted unifrac distance; (c) is based on unweighted unifrac distance. B30-O and Y30-O stand for original soil samples, and B30-T and Y30-T stand for soil samples collected on day 65.

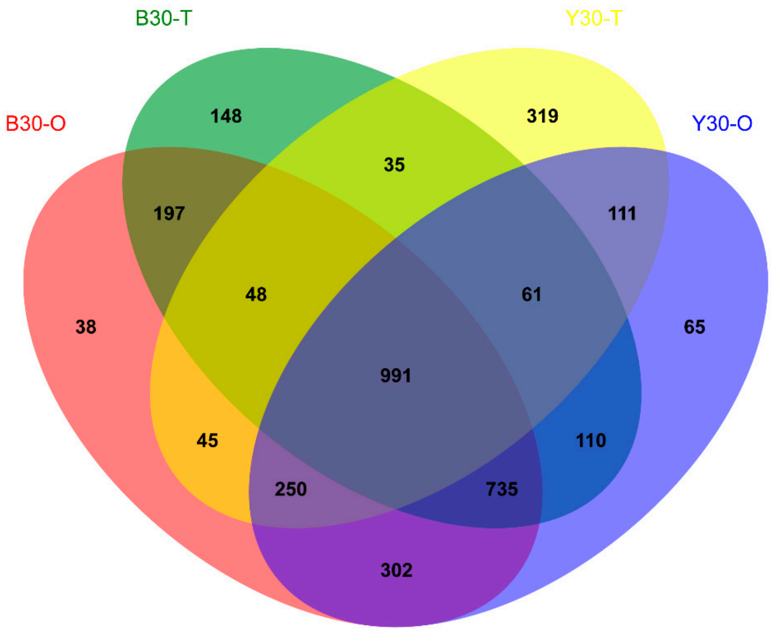

Figure S4. Venn diagram of the specific and shared OTUs in all soil samples. B30-O and Y30-O stand for original soil samples, B30-T and Y30-T stand for soil samples collected on day 65.
